# BSE-associated Prion-Amyloid Cardiomyopathy in Primates

**DOI:** 10.3201/eid1906.120906

**Published:** 2013-06

**Authors:** Susanne Krasemann, Giulia Mearini, Elisabeth Krämer, Katja Wagenführ, Walter Schulz-Schaeffer, Melanie Neumann, Walter Bodemer, Franz-Josef Kaup, Michael Beekes, Lucie Carrier, Adriano Aguzzi, Markus Glatzel

**Affiliations:** University Medical Center Hamburg-Eppendorf, Hamburg, Germany (S. Krasemann, G. Mearini, E. Krämer, M. Neumann, L. Carrier, M. Glatzel);; Robert Koch Institute, Berlin, Germany (K. Wagenführ, M. Beekes);; University Hospital Göttingen, Göttingen, Germany (W. Schulz-Schaeffer);; German Primate Center, Göttingen (W. Bodemer, F.-J. Kaup);; University of Zurich, Zurich, Switzerland (A. Aguzzi)

**Keywords:** BSE, cardiomyopathy, prions, PrP^Sc^, vCJD, variant Creutzfeldt-Jakob disease, bovine spongiform encephalopathy, primates

## Abstract

Prion amyloidosis occurred in the heart of 1 of 3 macaques intraperitoneally inoculated with bovine spongiform encephalopathy prions. This macaque had a remarkably long duration of disease and signs of cardiac distress. Variant Creutzfeldt-Jakob disease, caused by transmission of bovine spongiform encephalopathy to humans, may manifest with cardiac symptoms from prion-amyloid cardiomyopathy.

Human prion diseases are progressive neurologic disorders that include sporadic, genetic, and acquired forms of Creutzfeldt-Jakob disease (CJD) (*1*). A key step in disease initiation is conversion of PrP^C^ into PrP^Sc^, which is partially resistant to proteolytic digestion and an essential part of prion infectivity. Transmission of bovine spongiform encephalopathy (BSE) to humans has led to a novel form of acquired CJD, termed variant CJD (vCJD) (*2*). The pathogenesis of vCJD differs substantially from sporadic CJD with remarkable colonization of non–central nervous system regions with infectious prions and PrP^Sc^ (*3*).

Although risk reduction measures have been introduced to limit transmission from BSE-diseased cattle to humans, vCJD has occurred in several hundred instances (www.eurocjd.ed.ac.uk). Most clinically affected vCJD patients are homozygous for methionine on polymorphic codon 129 on the gene coding PrP (*PRNP*), and the clinical presentation of vCJD in these patients is uniform (*4*). The occurrence of atypical clinical features in persons with vCJD that encodes methionine and valine on *PRNP* codon 129 and human-to-human transmission of vCJD through blood transfusion have raised concern about atypical clinical features and alternative distribution of PrP^Sc^ in vCJD (*5*). We report on the novel clinicopathologic characteristics of vCJD as prion-amyloid cardiomyopathy in 1 of 3 macaques inoculated with BSE.

## The Study

**Figure 1:**
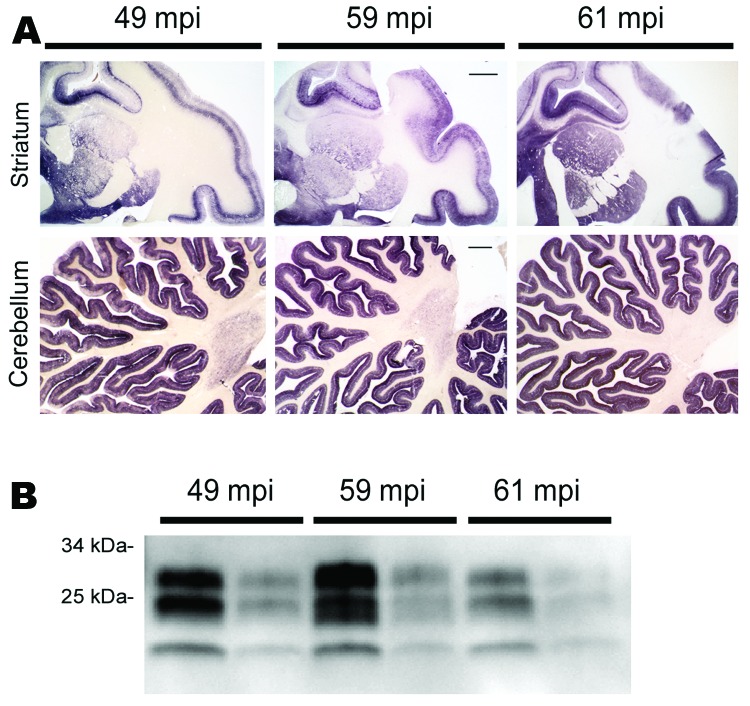
PrP^Sc^ distribution and content in brain of bovine spongiform encephalopathy (BSE)–infected rhesus macaques. A) Paraffin-embedded tissue blot of striatum and cerebellum show a typical BSE-like deposition pattern of PrP^Sc^ with no differences between individual BSE-diseased monkeys at 49, 59, and 61 months postinoculation (mpi). Scale bars = 1 mm. B) Western blot analysis for PrP^Sc^ in brain of BSE-infected monkeys with incubation times of 49, 59, and 61 mpi. PrP^Sc^-type is as expected for BSE prions, and no major differences in PrP^Sc^ load were detected. All samples were proteinase K–digested; loading amount was 0.5 and 0.1 mg fresh wet tissue for each sample

In 2002, three rhesus macaques were inoculated with BSE intraperitoneally (10 mL of a 10% homogenate of brain from BSE-diseased cattle). As controls, 2 rhesus macaques received saline (10 mL) and 1 was untreated. All procedures involving rhesus macaques were performed at the Institute of Neuropathology, University Medical Center Hamburg-Eppendorf (Hamburg, Germany), in accordance with the German Animal Welfare Act and the Council Directive 86/609/EEC (Permit 33.42502/08–08.02 LAVES, Lower Saxony, Germany). Animals were observed for clinical signs of prion disease and, when signs of terminal prion disease became evident, were euthanized and underwent autopsy. In all 3 BSE-challenged macaques and none of the controls a progressive neurologic disease developed 49, 59, and 61 months postinoculation. Examination of brain by using hematoxylin and eosin staining showed typical neuropathologic features of vCJD (data not shown) and abundant deposits of PrP^Sc^ in the cortex, basal ganglia, and cerebellum in paraffin-embedded tissue blots performed as described by using 12F10 monclonal antiprion antibody (*6*) (Figure 1, panel A). The mobility of the unglycosylated PrP^Sc^ band and the glycoform ratio of proteinase K–digested PrP^Sc^ were similar to those in BSE when assessed by Western blot analysis by using monoclonal POM-1 antiprion antibody as described (*7*) (Figure 1, panel B).

**Figure 2:**
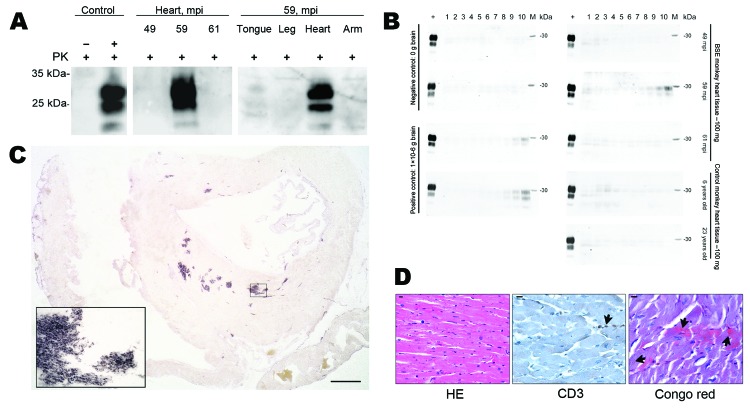
Abundant PrP^Sc^ in heart of 1 bovine spongiform encephalopathy (BSE)–infected rhesus macaque. A) In sodium phosphotungstic acid precipitation of PrP^Sc^, followed by Western blotting, highly abundant PrP^Sc^ was demonstrated in the heart of 1 BSE-infected primate. In this monkey, only the heart contained PrP^Sc^. Controls include cardiac muscle spiked with minimal amounts brain of a healthy (–) and prion-diseased (+) primate. All analyses were prepared from 50 mg of tissue except the heart of 1 monkey 59 months postinoculation (mpi) (20 mg). PK, proteinase K. B) In protein-misfolding cyclic amplification, PrP^Sc^ was amplified only from the heart of 1 monkey 59 months postinoculation (mpi). As a positive control, brain tissue from a BSE-diseased monkey was used, and tissue from an uninfected control monkey served as a negative control. PK–digested hamster PrP^Sc^ (263 K) served as loading and digestion control for PrP^Sc^. C) Paraffin-embedded tissue blotting of the entire heart of the 59 mpi monkey showed abundant deposition of PrP^Sc^, mainly in the septum of the heart. Inset confirms the deposition pattern of PrP^Sc^ as amyloid. Scale bar = 0.25 mm. D) Histologic and immunohistochemical examination of heart tissue of the 59-mpi monkey by using hematoxylin and eosin (HE) staining and immunohistochemical staining against T-cell marker CD3 showed regularly configured cardiomyocytes and only single T-cells associated with blood vessels (arrow). Congo red staining showed Congo red–positive material in cardiomyocytes in a patch-like deposition pattern (arrows). Scale bar = 10 µm.

Besides lymphoreticular tissues, the muscular compartment is targeted by prions (*7*,*8*). Thus, we assessed presence of PrP^Sc^ in skeletal and heart muscle by Western blot analysis with sodium phosphotungstic acid precipitation for enrichment of PrP^Sc^ and protein misfolding cyclic amplification by using published protocols (*3*). We could not detect substantial amounts of PrP^Sc^ in skeletal muscle (Figure 2, panel A). One macaque showed abundant PrP^Sc^ (≈1/100 of PrP^Sc^ found in brain) in heart in Western blot and protein misfolding cyclic amplification (Figure 2, panels A, B). Paraffin-embedded tissue blot analysis of this heart showed PrP^Sc^ as amyloid, occupying considerable stretches of heart tissue, mainly in the septum (Figure 2, panel C), whereas no PrP^Sc^ could be seen in hearts of other macaques (data not shown). These findings were confirmed by strong Congo red–positive patch-like depositions in cardiomyocytes in the heart of this monkey (Figure 2, panel D). The primate with cardiac PrP^Sc^ showed the longest disease duration (4 months, compared with 4 weeks for other BSE-infected monkeys), signs of cardiac affection when assessed by relevant makers of cardiac hypertrophy and of cardiac distress–associated inflammation, and only this macaque showed clinical signs of fatigue and signs of cardiac distress (i.e., venous congestion) on autopsy (Technical Appendix, Table). Histologic examination of heart tissue with hematoxylin and eosin staining and immunohistochemical stainings against B and T cells (CD20 [not shown] and CD3) did not provide evidence for toxic cardiomyopathy (i.e., fibrosis or vacuolization), nor did we find signs of inflammatory reaction (Figure 2, panel D).

**Table:** Characteristics of 3 rhesus macaques in study of BSE-associated prion-amyloid cardiomyopathy*

Primate	Age at inoculation	Time to clinical disease, mo	Disease duration, wk	Cardiac PrP^Sc^	Signs of cardiac distress at autopsy
BSE inoculated	8 y	49	4	Neg	Neg
	5 y	59	18	Pos	Pos
	1 y	61	4	Neg	Neg
Control	8 mo	NA	NA	Neg	Neg
	17 y	NA	NA	Neg	Neg
	19 y	NA	NA	Neg	Neg

*BSE, bovine spongiform encephalopathy; Neg, negative; Pos, positive; NA, not applicable.

## Conclusions

Although the vCJD epidemic is declining, considerable concern exists that clinical characterastics of vCJD will shift. The most important genetic risk factor for development of vCJD is homozygosity for methionine on *PRNP* codon 129, and all but 1 patient with clinical vCJD carry this polymorphism (*5*). Thus, future cases of vCJD with longer incubation times are likely to comprise more patients with alternative codon 129 polymorphisms than methionine homozygosity. Data from rodent experiments indicate that clinical features of vCJD may differ in these patients (*9*). Thus, the next decades may see a shift in vCJD phenotypes. Further uncertainty for atypical cases in humans results from the possibility of secondary transmission of vCJD through blood products from subclinical carriers, which may lead to development of nonclassical vCJD phenotypes (*5*).

We showed that BSE infection of primates may occur as prion-amyloid cardiomyopathy. Because prion-amyloid cardiomyopathy developed in only 1 of 3 macaques, host-encoded factors, such as genetic makeup, probably influence development of this cardiac phenotype. All macaques are homozygous for methionine on *PRNP* codon 129; thus, prion-amyloid cardiomyopathy cannot be related to polymorphic codon 129 in our study (*10*). Cardiac involvement has been observed in a patient with sporadic CJD and is prominent in prion-diseased mice expressing PrP^C^ lacking its membrane anchor (*11*,*12*). We considered the possibility that preexisting pathology, such as spontaneous cardiomyopathy or inflammation of the heart, might have contributed to cardiac PrP^Sc^, and the fact that we did not find any evidence for toxic cardiomyopathy or inflammation in the primate does not exclude this possibility. Because the macaque with abundant PrP^Sc^ deposition in heart had longer disease duration, it is also possible that longer disease duration, which favors centrifugal spread of prions to peripheral tissues, contributed to cardiac affection in this primate (*7*). Peripheral deposition of PrP^Sc^ in vCJD is well studied (*3*). We were surprised by the amount and deposition type of PrP^Sc^ in heart, reaching 1/100 of the amount seen in brain and deposited as amyloid across large stretches of heart tissue. Skeletal muscle of prion-diseased patients and nonhuman primates routinely harbor minimal amounts of PrP^Sc^ (<1/1000 that found in brain), and PrP^Sc^ in muscle is virtually impossible to detect by in situ methods (*6*,*8*,*13*). To our knowledge, PrP^Sc^ has not been detected in heart of vCJD-diseased persons or in patients with systemic amyloidosis, although primates orally exposed to BSE show very low amounts of cardiac PrP^Sc^ (*8*,*14*,*15*). The lack of cardiac PrP^Sc^ in vCJD may result from small cohorts investigated. Because the spectrum of vCJD is likely to change, broad application of current clinical criteria for vCJD in clinical practice may lead to underreporting of vCJD, missing atypical cases of vCJD.

In conclusion, we showed that BSE-infection of primates may lead to prion-amyloid cardiomyopathy. These data should be considered when vCJD surveillance is conducted.

Technical Appendix RNA isolation method and supplementary results.
