# Supplementary material for: BSE-associated Prion-Amyloid Cardiomyopathy in Primates
Source: Emerg Infect Dis. 2013 Jun;19(6):985–8. doi: 10.3201/eid1906.120906 (PMC3713817; doi:10.3201/eid1906.120906)
Supplement: Technical Appendix — RNA isolation method and supplementary results. [file 12-0906-Techapp-s1.pdf]

Article DOI: http://dx.doi.org/10.3201/eid1906.120906

BSE-associated Prion-Amyloid Cardiomyopathy in Primates

Technical Appendix

RNA Isolation and Analysis

Total RNA was extracted from 50 mg of heart tissue by using the SV Total RNA Isolation System (Promega, Madison, WI, USA) according to the manufacturer’s protocol. However, to denaturate PrP^{Sc}, lysis buffer containing 4 M guanidinium thiocyanate was added, and tissue homogenate was incubated 24 h at room temperature before clearing of debris by centrifugation and further processing as described in the protocol. The reverse transcription was performed by using the First strand cDNA synthesis kit (Thermo-Fisher Scientific former Fermentas, Schwerte, Germany) with oligo-dT primers from 100 ng RNA. Qualitative determination of macaque monkey mRNAs was performed with Phusion Hot Start II (Thermo Scientific former Finnzymes) polymerase and a touchdown PCR program between 60°–55°C. PCR products were visualized on 1%–2% agarose gels. Quantitative analysis of various macaca mRNAs was performed by real-time RT-PCR on a TaqMan ABI Prism 7900TH (Applied Biosystems, Foster City, CA, USA) machine using the Maxima SYBR Green/ROX qPCR Master Mix (Thermo Scientific former Fermentas), 900 nM primers and 1 µL cDNA (diluted 1:10). The mRNA levels of triplicates were analyzed with the comparative C_t method ($2^{-\Delta\Delta Ct}$) by using GAPDH as endogenous control. The specific primer pairs used are listed in the Table.

Technical Appendix Table. Primer sequences

Gene*	Primers
GAPDH F	5′-ATGTTCGTCATGGGTGTGAA-3′
GAPDH R	5′-TGAGTCCTTCCACGATACCA-3′
ANP F	5′-TCTCCACCATCAGTGTGAGC-3′
ANP R	5′-GGGCACGACCTCATCTTCTA-3′
TNFα F	5′-TCAGCCTCTTCTCCTTCCTG-3′
TNFα R	5′-CTTGGGGTTCGAGAAGATGA-3′

*F, forward; R, reverse

Results

To investigate whether BSE-infected monkeys differ from controls in mRNA levels indicative of cardiac hypertrophy and of cardiac distress–associated inflammation, we carried out RT-qPCR. Because many rhesus sequences are only predicted by automated computational analysis, the specificity of the primers was tested by sequencing of the corresponding PCR products. The mRNA levels of atrial natriuretic peptide (ANP), a marker of hypertrophy, were lower in all 3 prion-diseased animals than in controls (Technical Appendix Figure, panel A). The mRNA levels of tumor necrosis factor-α (TNF-α), a marker of cardiac-distress associated inflammation, was increased in the BSE-infected monkey, with cardiac PrP^{Sc} (Technical Appendix Figure, panel B).

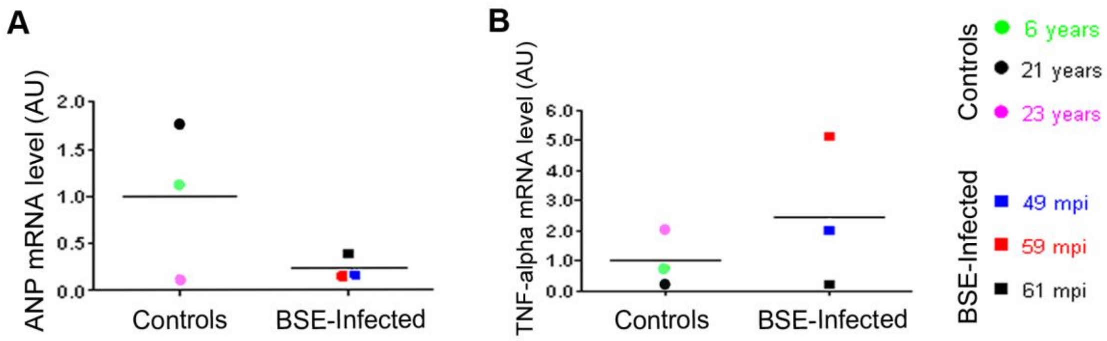

Technical Appendix Figure. Assessment of markers for hypertrophy and cardiac distress–associated inflammation in rhesus macaques. A, B) The mRNA levels of indicated marker proteins were determined in reverse transcription quantitative PCR with specific primers by using SyBR Green and GAPDH as endogenous control. Each sample was analyzed in triplicates. ANP, atrial natriuretic peptide; AU, arbitrary units.
